# Global Prevalence of Chronic Pain Among Cancer Survivors: A Systematic Review and Proportional Meta‐Analysis of Observational Studies

**DOI:** 10.1111/wvn.70122

**Published:** 2026-02-03

**Authors:** Pak Kit Wong, Lizhen Wang, Mu‐Hsing Ho, Chia‐Chin Lin

**Affiliations:** ^1^ School of Nursing, LKS Faculty of Medicine The University of Hong Kong Pokfulam Hong Kong SAR; ^2^ Alice Ho Miu Ling Nethersole Charity Foundation Professor in Nursing Tai Po Hong Kong SAR

**Keywords:** cancer survivors, chronic pain, prevalence, proportional meta‐analysis, systematic review

## Abstract

**Background:**

Cancer survivors often receive inadequate pain management, leading to impaired quality of life. Despite their importance, evidence on the global prevalence of chronic pain in cancer survivors remains insufficiently clear.

**Aims:**

The systematic review and proportional meta‐analysis aimed to estimate the pooled global prevalence of chronic pain among cancer survivors and to explore heterogeneity stratified by geographic region, cancer type, pain duration, and pain assessment tool.

**Methods:**

Five databases (PubMed, Embase, Cochrane Library, CINAHL, and China National Knowledge Infrastructure) were searched up to September 2024 for studies in English or Chinese. The review followed MOOSE and PRISMA guidelines with PROSPERO registration (CRDxx). Studies were included if they reported chronic pain prevalence in cancer survivors using validated instruments or solely reported chronic pain. Two reviewers independently screened studies, extracted data, and assessed quality using the JBI Critical Appraisal Checklist for Analytical Cross‐Sectional Studies. Pooled prevalence and 95% prediction intervals were calculated using a random‐effects model with Freeman–Tukey double arcsine transformation. Subgroup analysis was used to explore heterogeneity. Leave‐one‐out analysis explored robustness. Funnel plot and Egger's test were used to examine publication bias.

**Results:**

In total, 36 studies involving 39,806 participants were included. The pooled prevalence of chronic pain among cancer survivors was 41% (95% CI: 34%–49%) after testing robustness, with significant heterogeneity (*I* ^*2*^ = 99.32%). Subgroup analysis indicated significant group differences in prevalence rates by geographic region, cancer type, and pain duration (all *p* < 0.001).

**Linking Evidence to Action:**

These findings stress the need for more extensive and tailored chronic pain management in current clinical practice. Additional research on chronic pain outcomes among pediatric cancer survivors, cancer populations within Africa and South America, and those with cancer types other than breast cancer is needed.

**Trail Registration:**

PROSPERO Registration: CRD42024597090.

## 1 Background

Globally, there were approximately 20 million new cancer diagnoses, while cancer developed in around one in every five people in their lifetime (Bray et al. 2024). With the advances in treatment modalities, the 5‐year net survival for most cancers has risen worldwide, resulting in a steadily growing population of survivors (Allemani et al. 2018). It is known that pain can have a detrimental impact on the quality of life of cancer survivors. A previous study showed a significant negative correlation between quality of life and pain variables, including pain intensity, pain distress, and pain interference among the cancer population (Rodriguez et al. 2019). A total of 33.75% of long‐term breast cancer survivors experienced moderate to severe pain, and individuals with chronic pain were more likely to have cancer‐related fatigue, lower physical activity levels, and mood disturbances (Alvarez‐Salvago et al. 2025). In a cohort study with a median follow‐up of 141 months, chronic pain in cancer patients was reported to be associated with an increased mortality rate (Zhang and Guo 2025). With the predicted 77% increase in global cancer incidence from 2022 to 2050 (Bray et al. 2024), the number of cancer survivors experiencing pain is expected to rise over the next two decades.

According to the eleventh revision of the International Classification of Diseases (ICD‐11), the definition of chronic pain is “persistent or recurrent pain lasting longer than 3 months” (Treede et al. 2015, 1004). The ICD‐11 also emphasizes that cancer treatments, such as chemotherapy, radiotherapy, and surgery, have the potential to lead to chronic post‐cancer treatment pain (Bennett et al. 2019). Due to the diversity of anticancer treatments, chronic post‐cancer treatment pain can be attributable to various treatment modalities (Bennett et al. 2019). Additionally, it is common for cancer survivors to undergo long‐term anticancer treatments that last for more than 3 months (Park et al. 2022). Hence, in this review, chronic pain in cancer survivors refers to pain persisting for at least 3 months in individuals who are either currently receiving or have completed any form of cancer treatment. In previous literature reporting the prevalence of chronic pain in both the general population and cancer survivors in the United States, the severity of the chronic pain was not presented; instead, the focus was on functional limitations or high‐impact chronic pain, which refers to chronic pain that severely interfered with an individual's life or work activities (Dahlhamer et al. 2018; Jiang et al. 2019; Yong et al. 2022). The prevalence of high‐impact chronic pain among cancer survivors was 16.1%, higher than the 8% observed in the general population in the United States (Dahlhamer et al. 2018; Jiang et al. 2019). In view of the rapid increase in global cancer incidence and the significantly detrimental impacts of chronic pain on cancer survivors, multiple guidelines have been developed for the management of pain among cancer survivors (Fallon et al. 2018; Swarm et al. 2019; World Health Organization 2018).

However, cancer survivors often receive inadequate pain management despite the well‐established guidelines (Roberto et al. 2022). Patient‐related barriers include a knowledge deficiency regarding pain management (Makhlouf et al. 2020). The same study reported that survivors are often concerned about the involvement of opioids in their pain management regimen, fearing addiction, tolerance, or adverse effects. Such misconceptions can lead to reluctance in reporting pain or poor adherence to prescribed opioids. Moreover, sociodemographic inequalities may contribute to the undertreatment of cancer pain. Survivors from low socioeconomic backgrounds face challenges in accessing survivorship care, including adequate cancer pain management (Stein et al. 2016). In fact, survivors were found to be more likely to experience pain in low‐income countries (Snijders et al. 2023). These obstacles should be overcome to preserve the quality of life among cancer survivors. Therefore, understanding the pooled prevalence of chronic pain across different geographic regions may aid in developing targeted policies and pain management strategies for cancer survivors.

Nevertheless, insufficient information exists regarding the global prevalence of chronic pain among cancer survivors and the underlying risk factors involved, while most studies did not clearly differentiate between acute and chronic pain (Jiang et al. 2019). Evenepoel et al. (2022) assessed the prevalence of pain in cancer patients during or within 3 months after curative cancer treatment in Europe, North America, Oceania, and Asia, based on only a total of 12 studies. Although Snijders et al. (2023) included 444 studies of patients reporting pain from all phases of cancer care, all patients reporting pain post‐curative cancer treatment were included, regardless of the pain's duration. Consequently, the review did not distinguish clearly between acute and chronic pain in cancer survivors. Based on previous literature, the global prevalence of chronic pain in cancer survivors remains insufficiently understood. Given the chronic nature of cancer survivorship, it is important to explore chronic pain among cancer survivors. Therefore, the aim of this study is to provide a snapshot of chronic pain prevalence among cancer survivors.

## 2 Methods

This systematic review followed the guidelines from Meta‐analysis of Observational Studies in Epidemiology (MOOSE) group (Stroup et al. 2000) and reported in accordance with Preferred Reporting Items for Systematic reviews and Meta‐Analyses (PRISMA) (Page et al. 2021). The PROSPERO registration number for this review is CRD 42024597090.

### 2.1 Search Strategy

A literature search was conducted up to September 30, 2024 across five databases, namely PubMed, Embase, Cochrane Library, CINAHL, and China National Knowledge Infrastructure (CNKI). A combination of the keywords ‘chronic pain’ and ‘neoplasms’ was used. For PubMed, MeSH terms were specifically used to enhance the precision of the search results. The detailed search strategy is presented in Appendix S1.

### 2.2 Inclusion and Exclusion Criteria

Eligibility of the selected studies was based on the following criteria: (i) observational studies with cross‐sectional design reporting the prevalence of chronic pain, either only mentioning chronic pain or defining pain as lasting ≥ 3 months; (ii) target population consisting of cancer survivors; and (iii) written in English or Chinese language. Studies were excluded if they met any of the following criteria: (i) failure to specify the chronicity of pain; (ii) involvement of non‐cancer population; (iii) not an original study; and (iv) abstracts, commentaries, or letters to the editor. When encountering multiple studies utilizing the same dataset, the article with the largest sample size or providing the most comprehensive data was selected to avoid overcounting the prevalence of chronic pain.

### 2.3 Study Selection and Data Extraction

Two reviewers (PKW and MHH) screened the title and abstract of all retrieved records from the databases independently. Full text review of the remaining articles was then performed by the same reviewer. Data extraction of the eligible studies was conducted by the same reviewer and one research assistant independently. A Microsoft Excel 365 spreadsheet was created to enter the extracted data in a standardized format. The data entered were as follows: publication information (author and year), study location (country and corresponding geographical region), and study characteristics (design and sample size). Participant demographics encompassed male percentage and mean/median age. Clinical details covered cancer type, metastasis percentage, the defined duration, and the instrument used to assess pain. Functional limitations or high‐impact chronic pain were not extracted in this review due to the limited information in the included studies. The research team reached consensus through discussion when discrepancies arose.

### 2.4 Quality Appraisal

The risk of bias in the selected studies was assessed using the JBI Critical Appraisal Checklist for Analytical Cross‐Sectional Studies (Moola et al. 2020) by two reviewers. The checklist consists of eight questions, assessing sample representativeness, validity of exposure and outcome measures, identification and adjustment for confounding factors, and appropriateness of statistical analysis. The answer to each question is either ‘yes’, ‘no’, ‘unclear (NC)’ or ‘not applicable (NA)’. The quality score for each study was calculated based on the percentage of ‘yes’ responses to the checklist questions. Based on the quality score, each study was classified as low quality (< 33%), medium quality (33%–66%), or high quality (> 66%) (van Eikenhorst et al. 2017).

### 2.5 Statistical Analysis

All statistical analyses in this review were performed using Stata 18 software. We applied the Freeman‐Tukey double arcsine transformation of proportions to pool prevalence estimates, presenting them as percentages and their corresponding 95% confidence intervals (95% CI). Statistical heterogeneity was evaluated using the *I* ^*2*^ statistic. Since this review included studies with varied populations and geographical regions which could lead to variations in the reported prevalence rates, we anticipated a high degree of heterogeneity in the meta‐analysis. A random‐effects model was therefore used to pool prevalence estimates. We also conducted subgroup analyses based on geographical regions, cancer type, and pain duration. A sensitivity test was conducted using leave‐one‐out analysis to assess the effect of outliers. To assess publication bias or the small‐study effect, funnel plot and Egger's test results were evaluated. A trim‐and‐fill method was used to estimate the number of missing studies and adjust the pooled prevalence.

## 3 Results

### 3.1 Search Strategy

**FIGURE 1:**
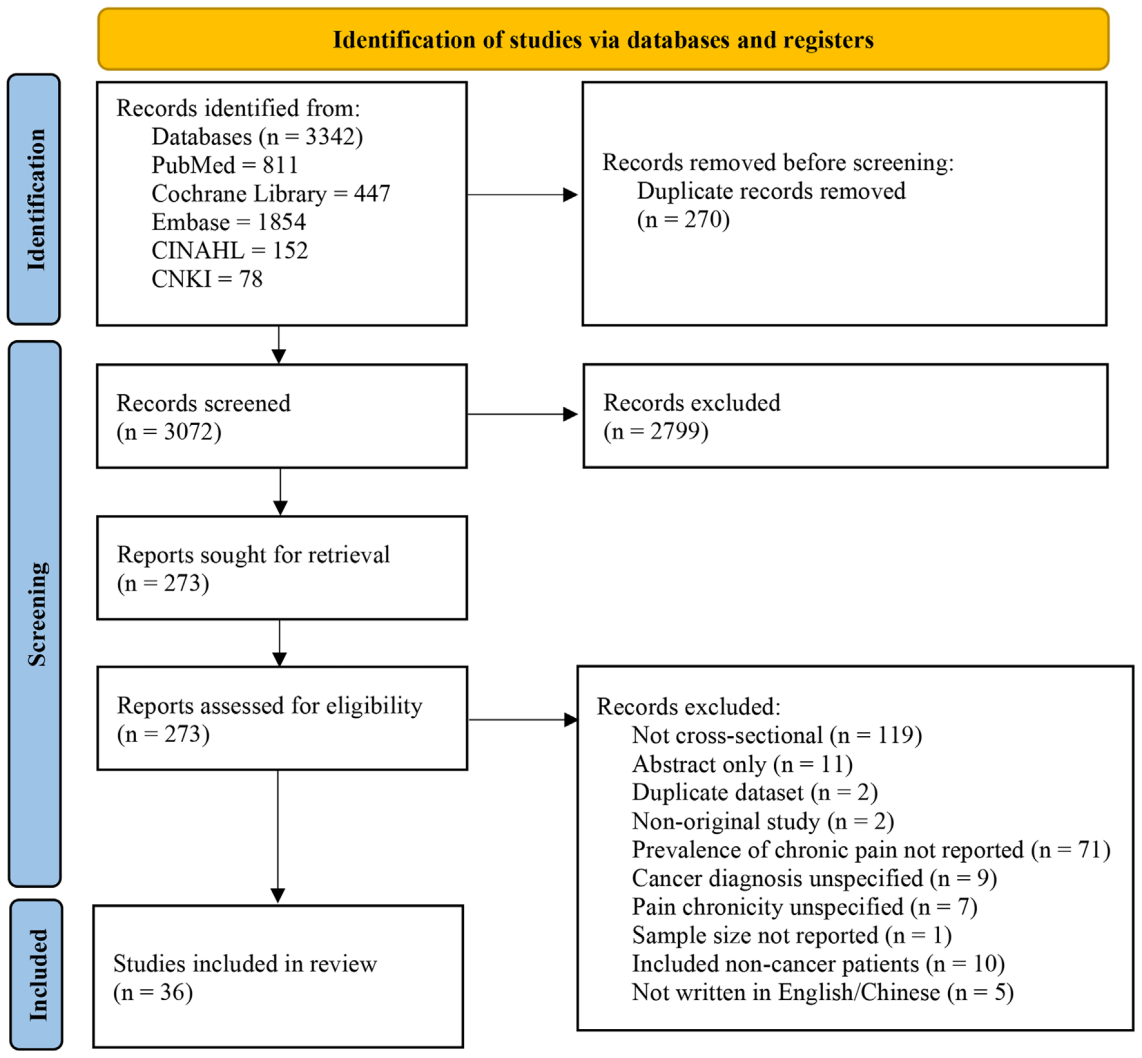
PRISMA flow diagram.

An initial search of the five databases resulted in a total of 3342 identified records (811 articles from PubMed, 447 articles from Cochrane Library, 1854 from Embase, 152 articles from CINAHL, and 78 articles from CNKI). After excluding 270 duplicates, 3072 records remained for title and abstract screening, of which 273 records were deemed eligible for full‐text review. Ultimately, 36 papers were included in this meta‐analysis. The PRISMA flow diagram (Figure 1) presents this study selection process in detail.

### 3.2 Study Characteristics

All 36 studies were cross‐sectional studies published between 1998 and 2024. This meta‐analysis included a total sample size of 39,806 participants, with the mean age of cancer survivors ranging from 12.8 to 71.5 years. The major geographical region where studies were conducted was Europe (*n* = 16, 44.4%), followed by North America (*n* = 9, 25%), Asia (*n* = 7, 19.4%), Oceania (*n* = 3, 8.33%), and Africa (*n* = 1, 2.78%). Most studies defined chronic pain as pain lasting more than 3 months (*n* = 16, 44.4%). The most common scale used by the studies to assess chronic pain was the Numeric Rating Scale (*n* = 14, 26.4%). The detailed study characteristics are presented in Table 1.

**TABLE 1:** Study characteristics of the included studies (*n* = 36).

Author, Year	Country, geographical region	Sample size	Male (%)	Mean age	Cancer type	Metastasis (%)	Pain duration	Instrument assessing pain
Akkaya et al. (2013)	Turkey, Europe	101	0%	50.8	Breast cancer	0%	≥ 3 months	VAS, FIQ
Andersen et al. (2012)	Denmark, Europe	2893	0%	CEF cohort: 49.8 ce + T cohort: 51.9	Breast cancer	0%	≥ 12 months	NRS
Bao et al. (2018)	US, North America	1280	0%	NR	Breast cancer	0%	NR	BPI
Björkstrand et al. (2024)	Sweden, Europe	276	17%	55.5	Mixed	NR	≥ 3 months	NR
Callegaro et al. (2015)	Italy, Europe	95	46.30%	55 (Median)	Retroperitoneal sarcoma	5.10%	NR	BPI
Cox‐Martin et al. (2020)	US, North America	1702	32.50%	NR	NR	NR	NR	NR
de la Rosa‐Díaz et al. (2022)	Spain, Europe	57	0%	NocP Group: 57.9 NeurFP Group: 56.1 No Pain Group: 63.1	Breast cancer	NR	≥ 1.5 years	LANSS, CSI, Spanish version of PCS
Donaldson et al. (2024)	New Zealand, Oceania	59	0%	NR	Breast cancer	0%	≥ 3 months	NR
Duarte et al. (2023)	Sweden, Europe	246	17.90%	55.8	Mixed	NR	≥ 3 months	BPI, NRS, CPAQ‐8
Dugué et al. (2022)	France, Europe	296	77.40%	NR	Head and neck cancer	6.42%	≥ 3 months	A seven‐point Likert‐type intensity pain scale
Edmond et al. (2017)	US, North America	350	0%	58.29	Breast cancer	0%	≥ 6 months	VAS, BPI
Feddern et al. (2015)	Denmark, Europe	1369	60%	65	Rectal cancer	0%	NR	BDDPQ, NRS
Hamood et al. (2018)	Israel, Asia	410	0%	With chronic pain: 63.8 Without chronic pain: 68.9	Breast cancer	0%	≥ 3 months	NRS
Hernández et al. (2023)	Spain, Europe	40	75%	59.95	Head and neck cancer	5%	NR	BPI, WPI
Høimyr et al. (2012)	Denmark, Europe	350	47.10%	60.9	Cutaneous melanoma	4.86%	≥ 3 months	NRS, DN4, PCS, Danish version of NPSI
Janah et al. (2020)	France, Europe	4093	37.40%	NR	Mixed	NR	≥ 3 months	DN4
Kaur et al. (2018)	India, Asia	215	0%	47.4	Breast cancer	0%	≥ 3 months	VAS, NPSI
Koczwara et al. (2023)	Australia, Oceania	177	0%	60.1 (Median)	Breast cancer	4.52%	NR	NR
Kurita et al. (2012)	Denmark, Europe	14,925	NR	NR	NR	NR	≥ 6 months	NRS
Lim (2018)	US, North America	157	0%	55.3	Breast cancer	0%	NR	NR
Lovell et al. (2021)	Australia, Oceania	274	Cancer with Pain Group: 47% Cancer with No Pain Group: 47%	Cancer with Pain Group: 70.8 Cancer with No Pain Group: 71.5	NR	NR	NR	BPI, NRS
Lowery et al. (2013)	US, North America	99	54.50%	64.71	Colorectal cancer	12.10%	NR	BPI, NPQ‐SF
Manfuku et al. (2019)	Japan, Asia	140	0%	Without Pain: 54.5 With Pain: 58.2	Breast cancer	0%	≥ 3 months	BPI, CSI, PCS
Mejdahl et al. (2013)	Denmark, Europe	2411	0%	64 (Median)	Breast cancer	0%	≥ 3 months	NRS
Philpot et al. (2023)	US, North America	1676	55.90%	66	NR	NR	≥ 3 months	NR
Ren et al. (2022)	China, Asia	1790	0%	49.1	Breast cancer	0%	NR	NRS
Schou Bredal et al. (2014)	Norway, Europe	834	0%	56	Breast cancer	0%	≥ 3 months	NRS, BPI, S‐LANSS
Schrier et al. (2012)	Israel, Asia	80	0%	55.3	Breast cancer	0%	≥ 3 months	BPI, FIQ
Shabangu et al. (2023)	South Africa, Africa	44	0%	60.5 (Median)	Breast cancer	NR	≥ 3 months	BPI, DN4, South African version of PCS
Song et al. (2017)	US, North America	560	56%	NR	Chordoma	11.70%	NR	NR
Steyaert et al. (2016)	Belgium, Europe	128	0%	56.5	Breast cancer	NR	NR	NRS, ID‐P
Tasmuth et al. (1998)	Finland, Europe	467	0%	MRM group: 61 BCT group: 57	Breast cancer	6.42%	NR	VAS, Finnish McGill Pain Questionnaire
Tatum et al. (2024)	US, North America	188	35.60%	63.72	Mixed	NR	NR	NRS
Williamson Lewis et al. (2021)	US, North America	579	52.30%	12.8	Mixed	NR	NR	NR
Zhumaliyeva et al. (2016)	Kazakhstan, Asia	374	19%	56	Mixed	0%	NR	NRS, NPQ‐C
Zuo et al. (2024)	China, Asia	1071	71.30%	50.6	Head and neck cancer	32.70%	≥ 3 months	NRS, Chinese version of LANSS

Abbreviations: BCT, breast‐conserving treatment; BDDPQ, Brief Descriptive Danish Pain Questionnaire; BPI, Brief Pain Inventory; CE + T, cyclophosphamide and epirubicin + docetaxel; CEF, cyclophosphamide, epirubicin and fluorouracil; CPAQ‐8, Chronic Pain Acceptance Questionnaire 8; CSI, Central Sensitization Inventory; DN4, Neuropathic Pain Diagnostic Questionnaire; FIQ, Fibromyalgia Impact Questionnaire; ID‐P, ID Pain questionnaire; LANSS, Leeds Assessment of Neuropathic Symptoms and Signs; MRM, modified radical mastectomies; NeurFP, pain with neuropathic features; NocP, nociceptive pain; NPQ‐C, Neuropathic Pain Questionnaire for Cancer Patients; NPQ‐SF, Neuropathic Pain Questionnaire‐Short Form; NPSI, Neuropathic Pain Symptom Inventory; NR, not reported; NRS, Numerical Rating Scale; PCS, Pain Catastrophizing Scale; S‐LANSS, self‐report version of the Leeds Assessment of Neuropathic Symptoms and Signs; VAS, Visual Analogue Scale; WPI, Widespread Pain Index.

### 3.3 Quality Appraisal

The risk of bias in all 36 studies was assessed using the JBI Critical Appraisal Checklist for Analytical Cross‐Sectional Studies. All of them were deemed high quality (*n* = 30, 83.3%) or medium quality (*n* = 6, 16.7%), with no studies being excluded due to low‐quality classification. The detailed assessment summary is presented in Table 2.

**TABLE 2:** Quality assessment of included studies using the JBI Critical Appraisal Checklist for Analytical Cross‐Sectional Studies.

Author, year	Q1	Q2	Q3	Q4	Q5	Q6	Q7	Q8	Quality assessment score
Akkaya et al. (2013)	Y	Y	Y	Y	N	N	Y	Y	75% (High)
Andersen et al. (2012)	Y	Y	Y	Y	Y	Y	Y	Y	100% (High)
Bao et al. (2018)	Y	Y	Y	Y	Y	Y	Y	Y	100% (High)
Björkstrand et al. (2024)	Y	Y	NC	N	N	N	N	Y	37.5% (Medium)
Callegaro et al. (2015)	Y	Y	Y	Y	Y	Y	Y	Y	100% (High)
Cox‐Martin et al. (2020)	Y	Y	N	N	Y	Y	N	Y	62.5% (Medium)
de la Rosa‐Díaz et al. (2022)	Y	Y	N	N	N	N	Y	Y	50% (Medium)
Donaldson et al. (2024)	Y	Y	Y	Y	N	N	Y	Y	75% (High)
Duarte et al. (2023)	Y	Y	Y	N	Y	Y	Y	Y	87.5% (High)
Dugué et al. (2022)	Y	Y	Y	Y	Y	Y	N	Y	87.5% (High)
Edmond et al. (2017)	Y	Y	Y	Y	Y	N	Y	Y	87.5% (High)
Feddern et al. (2015)	Y	Y	Y	Y	Y	Y	Y	Y	100% (High)
Hamood et al. (2018)	Y	Y	Y	Y	Y	Y	Y	Y	100% (High)
Hernández et al. (2023)	Y	Y	Y	Y	Y	Y	Y	Y	100% (High)
Høimyr et al. (2012)	Y	Y	Y	Y	Y	Y	Y	Y	100% (High)
Janah et al. (2020)	Y	Y	Y	Y	Y	Y	Y	Y	100% (High)
Kaur et al. (2018)	Y	Y	Y	Y	N	N	Y	Y	75% (High)
Koczwara et al. (2023)	N	Y	Y	NC	Y	Y	Y	Y	75% (High)
Kurita et al. (2012)	Y	Y	Y	N	Y	Y	Y	Y	87.5% (High)
Lim (2018)	Y	Y	Y	Y	Y	Y	N	Y	87.5% (High)
Lovell et al. (2021)	Y	Y	Y	NC	Y	Y	Y	Y	87.5% (High)
Lowery et al. (2013)	Y	Y	Y	Y	N	N	Y	Y	75% (High)
Manfuku et al. (2019)	Y	Y	Y	Y	Y	Y	Y	Y	100% (High)
Mejdahl et al. (2013)	Y	Y	Y	Y	Y	Y	Y	Y	100% (High)
Philpot et al. (2023)	Y	Y	Y	Y	Y	Y	N	Y	87.5% (High)
Ren et al. (2022)	Y	Y	Y	Y	N	N	Y	Y	75% (High)
Schou Bredal et al. (2014)	Y	Y	N	Y	Y	Y	Y	Y	87.5% (High)
Schrier et al. (2012)	Y	Y	Y	Y	Y	Y	Y	Y	100% (High)
Shabangu et al. (2023)	Y	Y	Y	Y	N	N	Y	Y	75% (High)
Song et al. (2017)	Y	Y	N	N	N	N	N	Y	37.5% (Medium)
Steyaert et al. (2016)	Y	Y	Y	Y	Y	Y	Y	Y	100% (High)
Tasmuth et al. (1998)	Y	Y	Y	Y	Y	Y	Y	Y	100% (High)
Tatum et al. (2024)	Y	Y	Y	N	Y	Y	Y	Y	87.5% (High)
Williamson Lewis et al. (2021)	Y	Y	Y	Y	N	N	N	Y	62.5% (Medium)
Zhumaliyeva et al. (2016)	Y	Y	NC	NC	N	N	Y	Y	50% (Medium)
Zuo et al. (2024)	Y	Y	Y	Y	Y	Y	Y	Y	100% (High)

*Note:* Questions of the JBI Critical Appraisal Checklist for Analytical Cross‐Sectional Studies: Q1: Were the criteria for inclusion in the sample clearly defined? Q2: Were the study subjects and the setting described in detail? Q3: Was the exposure measured in a valid and reliable way? Q4: Were objective, standard criteria used for measurement of the condition? Q5: Were confounding factors identified? Q6: Were strategies to deal with confounding factors stated? Q7: Were the outcomes measured in a valid and reliable way? Q8: Was appropriate statistical analysis used?

Abbreviations: JBI, Joanna Briggs Institute; N, no; UC, unclear; Y, yes.

### 3.4 Prevalence of Chronic Pain

**FIGURE 2:**
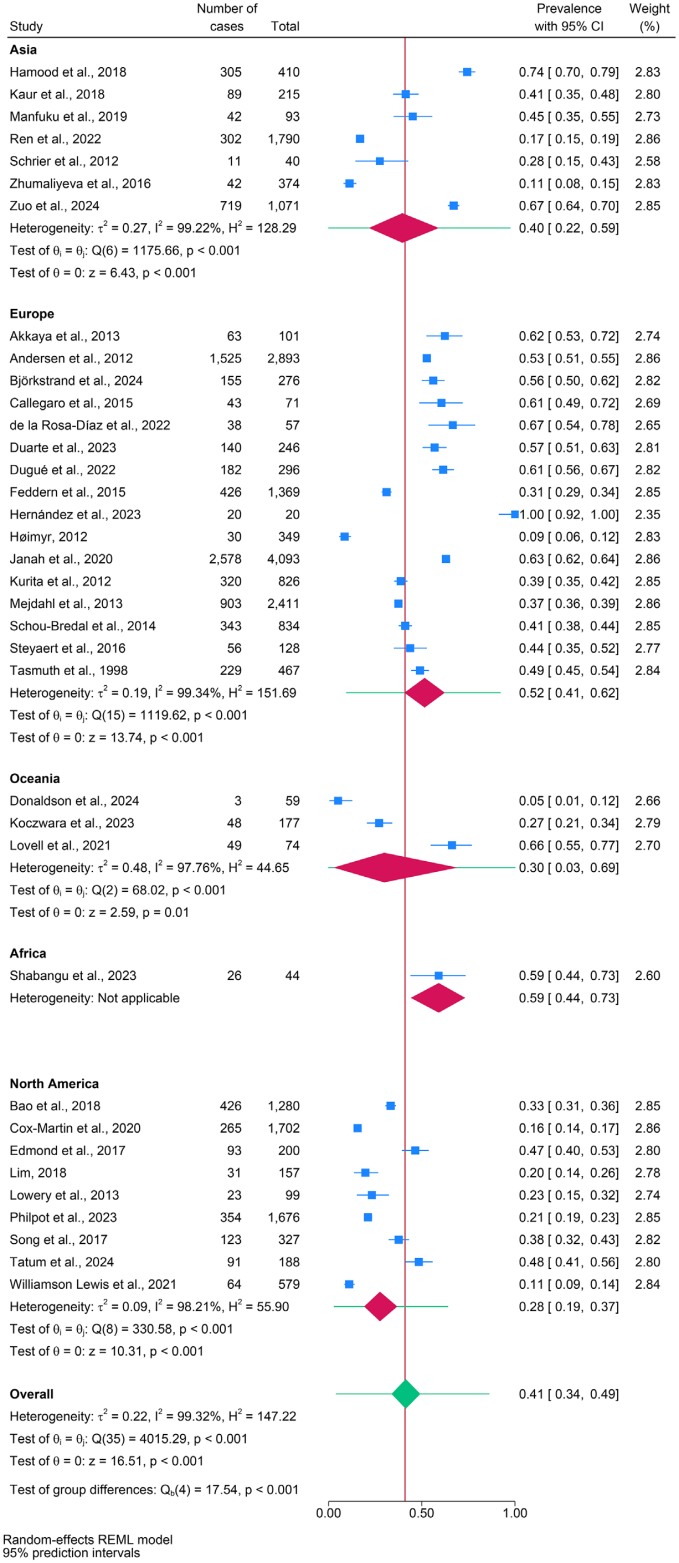
Forest plot of the overall pooled prevalence and estimates by geographical region.

**FIGURE 3:**
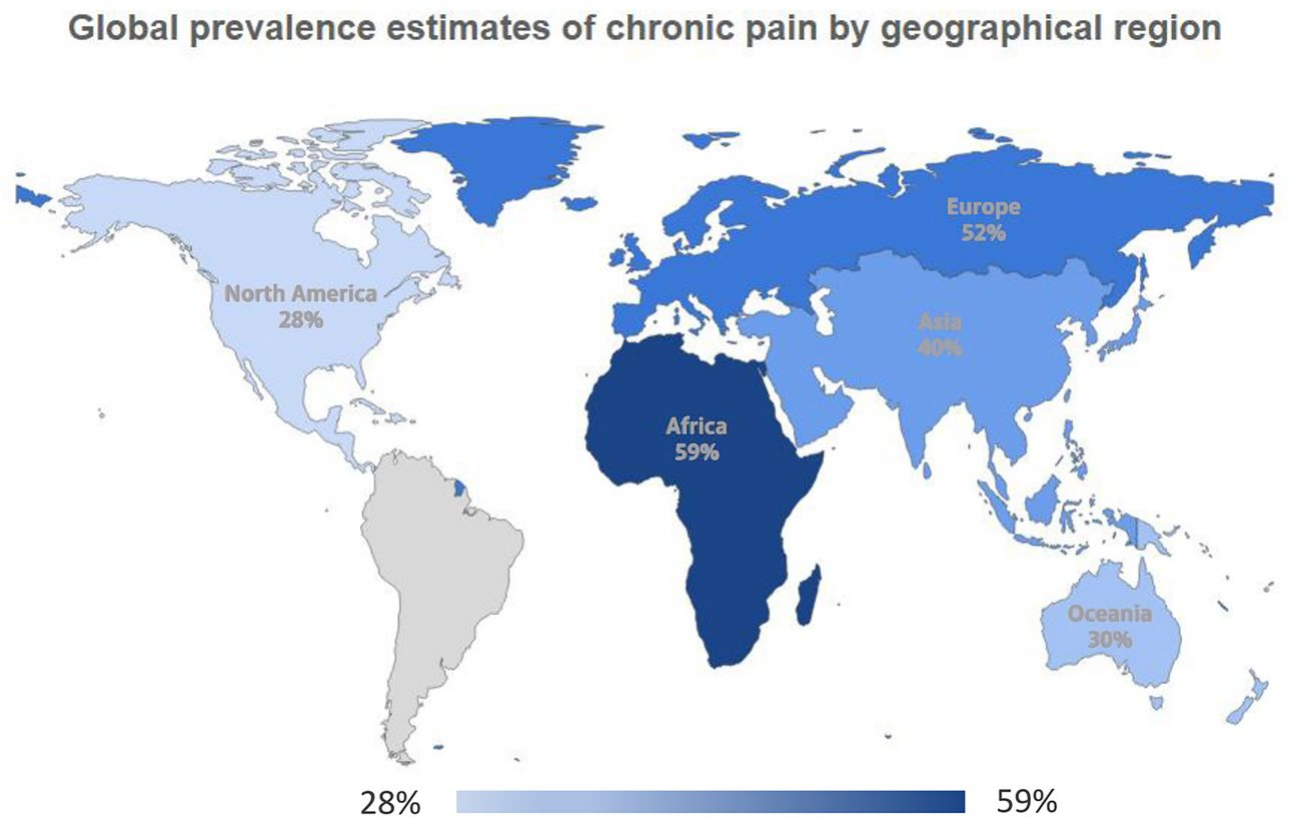
Global prevalence estimates of chronic pain by geographical region.

Figure 2 presents the forest plot of the overall pooled prevalence of the included 36 studies. The overall pooled prevalence rate of chronic pain was 41% (95% CI: 34%–49%). As anticipated, an *I* ^*2*^ value of 99.32% revealed considerable heterogeneity in prevalence estimates across the included studies. Figure 3 presents the global prevalence estimates of chronic pain by geographical region.

### 3.5 Subgroup Analysis

**FIGURE 4:**
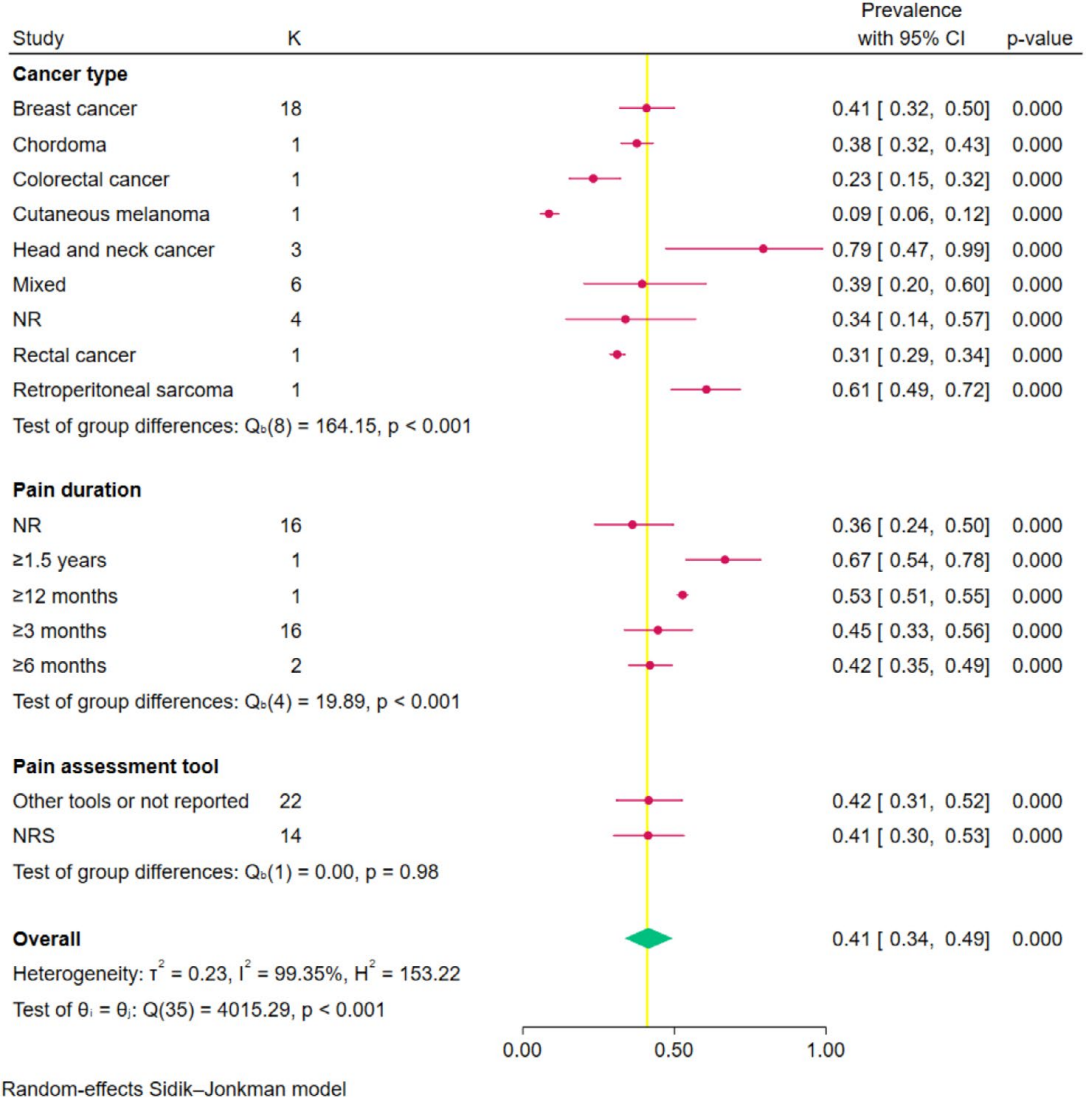
Forest plot of pooled prevalence estimates by cancer type and pain duration. NRS, Numerical Rating Scale.

Subgroup analysis indicated significant group differences in prevalence rates by geographic region, cancer type, and pain duration (all *p* < 0.001; Figures 2 and 4). The highest pooled prevalence rate of chronic pain by geographic region was Africa (59% [95% CI: 44%–73%]), followed by Europe (52%), Asia (40%), Oceania (30%), and North America (28%) (Figure 2). The pooled prevalence rates of chronic pain varied by cancer type: 41% (95% CI: 32%–50%) for breast cancer, 38% (95% CI: 32%–43%) for chordoma, 23% (95% CI: 15%–32%) for colorectal cancer, 9% (95% CI: 6%–12%) for cutaneous melanoma, 79% (95% CI: 47%–99%) for head and neck cancer, 39% (95% CI: 20%–60%) for mixed cancer types, 34% (95% CI: 14%–57%) for unspecified cancer types, 31% (95% CI: 29%–34%) for rectal cancer, and 61% (95% CI: 49%–72%) for retroperitoneal sarcoma (Figure 4). Regarding pain duration, prevalence rates were 36% (95% CI: 24%–50%) for unspecified duration, 67% (95% CI: 54%–78%) for pain persisting ≥ 1.5 years, 53% (95% CI: 51%–55%) for ≥ 12 months, 45% (95% CI: 33%–56%) for ≥ 3 months, and 42% (95% CI: 35%–49%) for ≥ 6 months (Figure 4). However, the pooled prevalence rate of chronic pain by different pain assessment tools was statistically insignificant (*p* = 0.98; Figure 4).

### 3.6 Leave‐One‐Out Analysis

**FIGURE 5:**
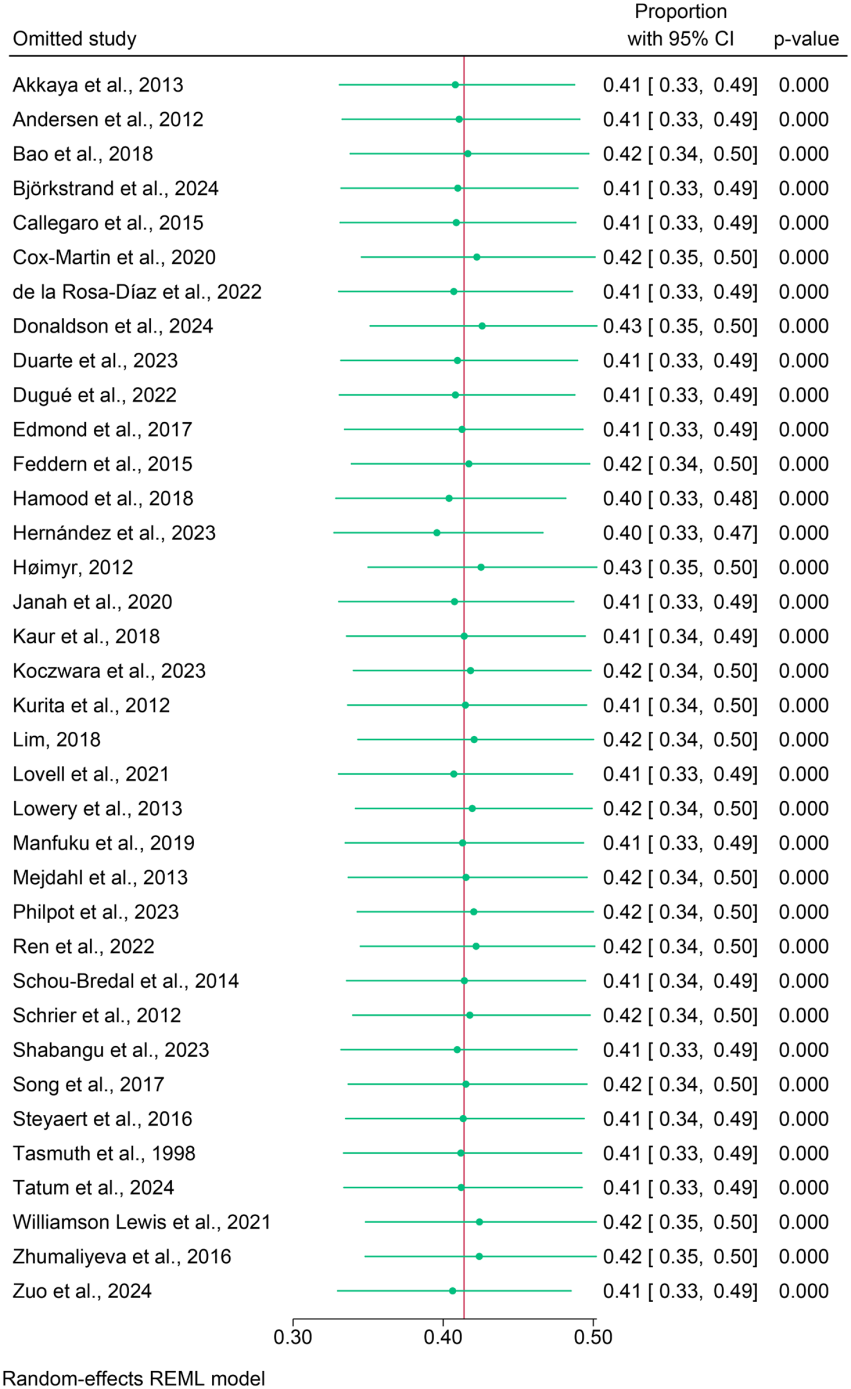
Leave‐one‐out analysis.

**FIGURE 6:**
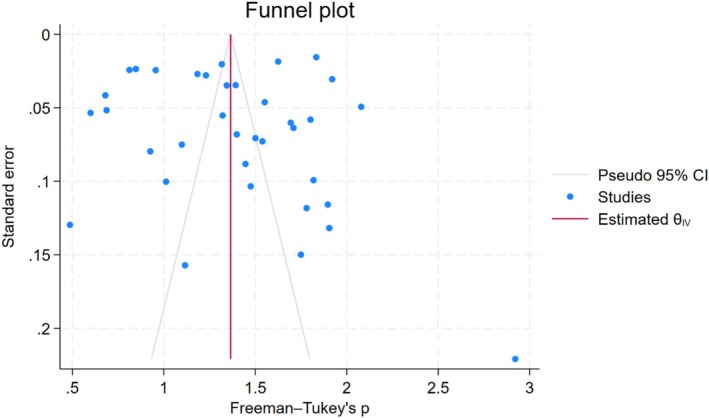
Funnel plot.

As presented in Figure 5, the leave‐one‐out analysis revealed that the overall pooled prevalence estimate of chronic pain remained stable regardless of which study was excluded. This indicates the absence of outliers that influenced the meta‐analytic result, confirming the robustness of the findings. Figure 6 presents the funnel plot for exploring publication bias. Egger's test revealed a statistically significant publication bias (*p* = 0.026). The trim‐and‐fill method showed that no additional studies needed to be added to balance the funnel plot with the same pooled results.

## 4 Discussion

This systematic review and proportional meta‐analysis aims to provide a snapshot of the overall prevalence of chronic pain among cancer survivors. Our pooled overall prevalence of chronic pain was 41% (95% CI: 34%–49%), despite considerable heterogeneity (*I* ^*2*^ = 99.32%). A previous meta‐analysis by Snijders et al. (2023) reported an overall pain prevalence of 44.5% (95% CI: 41.1%–47.9%) among cancer patients. Although not specific for chronic pain, this figure is similar to our study's findings. Notably, the 41% pooled prevalence in our review is substantially higher than the global 27.5% pain prevalence among the general population (Zimmer et al. 2022), highlighting that surviving cancer is often accompanied by persistent pain.

Our findings suggest the need for more extensive and tailored pain management in current clinical practice. In fact, a review of undertreated cancer pain revealed that 40.2% of cancer survivors received insufficient pharmacological pain control (Roberto et al. 2022). Additionally, a qualitative study exploring the experiences of cancer survivors with different cancer types in managing chronic pain after curative‐intent treatment found that healthcare providers often failed to recognize the presence of chronic pain, leading to its undertreatment (O'Regan et al. 2024). These findings underscore the importance of the skilled use of opioids as the primary pharmacological approach, as well as effective communication between patients and healthcare providers in managing cancer pain. Beyond pharmacology, invasive interventional analgesic therapies and non‐invasive methods are also recommended when appropriate to support a more personalized and integrative treatment plan (Mestdagh et al. 2023). The guideline by the European Society for Medical Oncology (ESMO) emphasizes the importance of continuous assessment of cancer pain, as it is crucial for the initiation of an individualized regimen for effective pain management (Fallon et al. 2018). Additionally, non‐invasive interventions such as hypnosis, music therapy, and acupuncture have been shown to effectively reduce pain in cancer patients. Meanwhile, therapies like massage and mind–body practices can provide relief from anxiety and depression associated with pain, further supporting a comprehensive approach to pain management (Deng 2019).

After conducting subgroup analysis, significant group differences were observed in chronic pain prevalence by geographic region (*p* < 0.001), cancer type (*p* < 0.001), and pain duration (*p* < 0.001). Contrary to the results by (Snijders et al. 2023), which concluded that Europe had a lower prevalence of cancer‐related pain than Asia, Africa, and South America, our results showed that Europe had the second‐highest chronic pain prevalence (52%; 95% CI: 41%–62%), following Africa (59%; 95% CI: 44%–73%), while North America exhibited the lowest prevalence (28%; 95% CI: 19%–37%). A survey found that high‐income countries were more likely to implement survivorship care practices, including routine symptom monitoring (Mollica et al. 2020). These practices facilitate the identification and reporting of ongoing symptoms by survivors, providing a possible explanation for this discrepancy. Notably, evidence also indicated that prostate cancer patients in Eastern Europe and Asia were less likely to use opioids, the mainstay of cancer pain management, compared to those in North America (Roydhouse et al. 2019), implying that more adequate pain management in North America might contribute to the lower prevalence of chronic pain among cancer survivors there.

Among the included cancer types, head and neck cancer survivors yielded the highest chronic pain prevalence rate (79%; 95% CI: 47%–99%). This heightened prevalence of chronic pain can be attributed to various factors: rich sensory innervation in the head and neck region, orofacial and cervical movements essential for daily functions, and the aggressive disease progression of head and neck cancer (Khawaja et al. 2021). It is worth noting that head and neck cancer survivors endure higher psychosocial distress when compared to those with other cancer types. Head and neck cancer, along with its treatment, can lead to disfigurement and functional impairments, imposing significant emotional and psychological challenges on its survivors. A study revealed that the prevalence of depression among head and neck cancer patients was 40.1%, nearly double that of patients with other cancers (Martinez et al. 2024).

Our review highlights gaps in future research. We found that half (50%) of the included studies assessed chronic pain among female breast cancer survivors specifically (*n* = 18). However, breast cancer only accounted for 11.6% of global cancer diagnoses worldwide in 2022 (Bray et al. 2024), which is disproportionate to the representation of breast cancer‐focused studies in our sample. This suggests an underrepresentation of chronic pain data for survivors of other cancer types. A similar issue was observed with age group and geographical region representation. Only one study (Williamson Lewis et al. 2021) in our sample (2.78%) reported prevalence data on chronic pain among pediatric patients. Similarly, only one included study (Shabangu et al. 2023) reported prevalence data on chronic pain in Africa. No studies in our sample reported prevalence data on chronic pain in South America. Future studies should prioritize underrepresented groups, such as pediatric survivors, populations within Africa and South America, and cancer types with less‐studied chronic pain outcomes.

### 4.1 Limitations

This review has several limitations. First, high heterogeneity was present in our pooled prevalence of chronic pain. As previously discussed, our meta‐analysis sampled cancer patients with diverse types of cancer as well as geographical regions. Moreover, variation in the defined time frame for chronic pain may also contribute to the considerable heterogeneity of our pooled prevalence. Therefore, our results should be interpreted with caution. Second, the use of unvalidated scales in assessing chronic pain by some of the included studies may have introduced measurement bias, potentially affecting the reliability of the reported prevalence rates. Third, the failure of some studies to specify the duration of chronic pain poses a threat to the internal validity of our findings. Fourth, as addressed earlier in the text, the predominance of breast cancer survivors in our sample may lead to the underrepresentation of other survivors, affecting the generalizability of our results. Fifth, the exclusion of non‐English or Chinese studies may have resulted in the omission of high‐quality data from other regions, potentially limiting the scope and representativeness of our findings. Finally, publication bias may exist, warranting cautious interpretation of the prevalence data despite the unchanged pooled results from the trim‐and‐fill method.

Still, the strengths of this review should not be overlooked. The utilization of five databases in our search strategy allowed us to include papers from a wide range of countries. Additionally, the use of an appraisal checklist indicated that all sampled studies were at low risk of bias. Leave‐one‐out analysis also suggested the absence of outliers among the included articles. Together, these methodological strengths contribute to the overall validity and reliability of our meta‐analytic findings.

### 4.2 Linking Evidence to Action

- The pooled global prevalence of chronic pain among cancer survivors is 41% (95% CI: 34%–49%), based on 36 studies including 39,806 participants, with significant heterogeneity.
- Significant differences in pain prevalence were found by geographic region, cancer type, and pain duration (all *p* < 0.001).
- These results highlight the need for improved, tailored pain management and further research on underrepresented populations, such as pediatric survivors and those from Africa and South America.
- The study emphasizes the importance of standardized pain assessment tools to improve consistency in future research and clinical practice.

## 5 Conclusion

This meta‐analysis of 36 cross‐sectional studies revealed a pooled chronic pain prevalence of 41% (95% CI: 34%–49%) among cancer survivors. Subgroup analysis showed significant differences in pain prevalence by geographic region, cancer type, and pain duration (all *p* < 0.001). These findings underscore the need for more extensive and tailored chronic pain management in current clinical practice. Furthermore, this study highlights the need for additional research on chronic pain outcomes among pediatric cancer survivors, populations within Africa and South America, and those with cancer types other than breast cancer.

## Funding

The authors have nothing to report.

## Conflicts of Interest

The authors declare no conflicts of interest.

## Supporting information


**Appendix S1:** Search strategies.

## Data Availability

The datasets generated during and/or analyzed during the current study are available from the corresponding author upon reasonable request.
