# Supplementary material for: Global Prevalence of Chronic Pain Among Cancer Survivors: A Systematic Review and Proportional Meta‐Analysis of Observational Studies
Source: Worldviews Evid Based Nurs. 2026 Feb 3;23(1):e70122. doi: 10.1111/wvn.70122 (PMC12869016; doi:10.1111/wvn.70122)
Supplement: Supplementary file 1 — Appendix S1: Search strategies. [file WVN-23-0-s001.docx]

**Supplementary material S1: Search Strategies**

1. PubMed: "Chronic Pain" [Mesh] AND "Neoplasms" [Mesh]
2. Cochrane Library: "Chronic Pain" AND "Neoplasms"
3. Embase: ("Chronic pain" and "Neoplasm").mp.
4. CINAHL: "Chronic pain" AND "Neoplasms"
5. CNKI: "Chronic pain" and "Neoplasms"
